# Editorial Comment on “Peritumoral Infiltration of Regulatory T Cells Reduces the Therapeutic Efficacy of Bacillus Calmette–Guérin Therapy for Bladder Carcinoma In Situ”

**DOI:** 10.1111/iju.70089

**Published:** 2025-05-02

**Authors:** Kimiharu Takamatsu

**Affiliations:** ^1^ Department of Medical Biochemistry and Biophysics Karolinska Institutet Stockholm Sweden; ^2^ Department of Urology Keio University School of Medicine Tokyo Japan

**Keywords:** BCG vaccine, bladder cancer, regulatory T cell, tumor microenvironment

Carcinoma in situ (CIS) of the bladder is an aggressive form of non‐muscle‐invasive bladder cancer (NMIBC) with a high potential for progression to muscle‐invasive bladder cancer (MIBC) and metastasis. Intravesical instillation of Bacillus Calmette–Guérin (BCG) immunotherapy is the standard treatment for high‐risk and intermediate‐risk papillary NMIBC, as well as for CIS. However, patients treated with BCG exhibit a recurrence rate of 40%–60% and a progression rate of 9.5%–23.4%, highlighting the need for optimized therapeutic strategies, including timely aggressive interventions such as radical cystectomy and novel immuno‐oncology agents [1]. Despite its widespread use, randomized controlled trials (RCTs) for BCG‐resistant CIS remain insufficient, and clear guidelines for oncological decision‐making and follow‐up scheduling are still lacking [2]. Establishing precise biomarkers is essential for the future of precision medicine.

In this study, Fukiage et al. investigated the relationship between FOXP3 + density, the FOXP3+/CD4+ cell density ratio within 20 μm of the lower edge of bladder CIS, and the response to BCG treatment [3]. The authors focused on FOXP3+ cells, a marker for regulatory T cells (Tregs), in close proximity to CIS cancer cells and proposed that Tregs enrichment adjacent to CIS could serve as a biomarker at the time of CIS diagnosis, prior to BCG instillation. While previous studies have associated Tregs with treatment resistance in BCG‐treated CIS [4], this study highlights the significant influence of peritumoral Tregs on oncological outcomes. Interestingly, in this cohort, metastases were observed exclusively in the high Tregs near CIS group, although the difference did not reach statistical significance. BCG treatment can lead to two distinct immune microenvironments: an immunosuppressive microenvironment, which is associated with poor prognosis, and an immune‐enhanced microenvironment, characterized by high CD8+ T cell infiltration, which correlates with favorable outcomes [5]. Although this study did not investigate the mechanisms by which Tregs adjacent to CIS interact with cancer cells during BCG treatment, their peritumoral distribution may serve as a surrogate biomarker for immune microenvironmental profiling for predicting resistance to BCG therapy and could inform the design of clinical trials.

Larger prospective cohort studies and further validation studies are required to clarify the role of Tregs in BCG resistance and determine whether their effects can be modulated through additional cancer immunotherapies. If validated, assessing Treg distribution could become a valuable tool in clinical urology.

## Author Contributions

**Kimiharu Takamatsu:** conceptualization, writing – original draft, writing – review and editing.

## Conflicts of Interest

The author declares no conflicts of interest.
